# Inflammatory and apoptotic remodeling in autonomic nervous system following myocardial infarction

**DOI:** 10.1371/journal.pone.0177750

**Published:** 2017-05-18

**Authors:** Chen Gao, Kimberly Howard-Quijano, Christoph Rau, Tatsuo Takamiya, Yang Song, Kalyanam Shivkumar, Yibin Wang, Aman Mahajan

**Affiliations:** 1 Division of Molecular Medicine, David Geffen School of Medicine, University of California at Los Angeles, Los Angeles, California, United States of America; 2 Department of Anesthesiology and Perioperative Medicine, David Geffen School of Medicine, University of California at Los Angeles, Los Angeles, California, United States of America; 3 UCLA Cardiac Arrhythmia Center, David Geffen School of Medicine, University of California at Los Angeles, Los Angeles, California, United States of America; University of Cincinnati College of Medicine, UNITED STATES

## Abstract

**Background:**

Chronic myocardial infarction (MI) triggers pathological remodeling in the heart and cardiac nervous system. Abnormal function of the autonomic nervous system (ANS), including stellate ganglia (SG) and dorsal root ganglia (DRG) contribute to increased sympathoexcitation, cardiac dysfunction and arrythmogenesis. ANS modulation is a therapeutic target for arrhythmia associated with cardiac injury. However, the molecular mechanism involved in the pathological remodeling in ANS following cardiac injury remains to be established.

**Methods and results:**

In this study, we performed transcriptome analysis by RNA-sequencing in thoracic SG and (T1-T4) DRG obtained from Yorkshire pigs following either acute (3 to 5 hours) or chronic (8 weeks) myocardial infarction. By differential expression and weighted gene co-expression network analysis (WGCNA), we identified significant transcriptome changes and specific gene modules in the ANS tissues in response to myocardial infarction at either acute or chronic phases. Both differential expressed genes and the member genes of the WGCNA gene module associated with post-infarct condition were significantly enriched for inflammatory signaling and apoptotic cell death. Targeted validation analysis supported a significant induction of inflammatory and apoptotic signal in both SG and DRG following myocardial infarction, along with cellular evidence of apoptosis induction based on TUNEL analysis. Importantly, these molecular changes were observed specifically in the thoracic segments but not in their counterparts obtained from lumbar sections.

**Conclusion:**

Myocardial injury leads to time-dependent global changes in gene expression in the innervating ANS. Induction of inflammatory gene expression and loss of neuron cell viability in SG and DRG are potential novel mechanisms contributing to abnormal ANS function which can promote cardiac arrhythmia and pathological remodeling in myocardium.

## Introduction

Myocardial ischemia induces changes in the neurohumoral control systems, including sympathoexcitation, that contribute to autonomic nervous system (ANS) imbalance and myocardial remodeling[1, 2] [3]. Cardiac ANS is comprised of the intrinsic and extrinsic networks of the cardiac neural circuits that closely control cardiac function [4–6]. The intrinsic cardiac ANS includes large number of cardiac ganglia forming synapses with the sympathetic and parasympathetic fibers throughout the heart[4, 7, 8]. The extrinsic cardiac ANS, including spinal cord, dorsal root ganglia and stellate ganglia, mediate connections between the heart and the central nervous system. Dynamic interactions between peripheral and central portion of the cardiac nervous system are critical for the maintenance of normal cardiac function [9, 10]. Imbalances in reflex processing within the peripheral and central systems are fundamental to progression of cardiac pathology, including the potential of sudden cardiac death (SCD) [11–13]. For example, there is an increase in basal excitability and remodeling of neurochemicals in the intrinsic cardiac nervous system as mediated by acetylcholine, catecholamine, and neuropeptides which increase myocardial sympathoexcitation and risk for cardiac arrhythmias. [14]. Neural remodeling has been demonstrated in the intrinsic cardiac ANS and extracardiac intrathoracic sympathetic ganglia, therefore it likewise may also be manifested throughout the extracardiac ANS including the spinal cord and dorsal root ganglia[15, 16].

In the diseased heart, there is evidence that abnormal autonomic function can lead to and exacerbate congestive heart failure, ventricular arrhythmias, and sudden cardiac death [1, 2] [3]. Clinically, inhibition of the sympathetic ANS through neural modulation techniques such as thoracic epidural anesthesia, spinal cord stimulation, and stellate ganglion sympathectomy, has been shown to be beneficial in treating ventricular arrhythmias[17–21]. In post-MI dogs, the incidence of ventricular fibrillation was decreased from 66% to zero by performing left stellate ganglionectomy[22]. In humans after MI, left stellate decentralization decreased the incidence of SCD from 21% to 2.7%[23]. Imbalance of the ANS has also been demonstrated to induce significant changes in atrial electrophysiology; and reducing autonomic innervation or signal outflow has shown benefits in reducing the incidence of spontaneous atrial arrhythmias[24–27]. In our earlier studies, we have utilized Yorkshire pigs as a model system to successfully demonstrate that modulation of the stellate ganglia and dorsal root afferents from the dorsal root ganglion (DRG) to the spinal cord can modulate ventricular sympathetic control and myocardial excitability[9, 28]. However, the molecular pathways through which this modulation occurs remain unknown.

Compared to the comprehensive focus on the molecular studies in the myocardium, our knowledge of the pathological remodeling in the cardiac ANS has been limited to few protein markers[29]. Myocardial infarction leads to sympathoexcitation and is a major trigger of lethal cardiac arrhythmias; however, the direct impact of myocardial infarction on alternations in molecular structure of the sympathetic ANS, especially on global level, is still underexplored. Therefore, the aim of this study is to compare the global molecular changes in DRG and SG following acute or chronic myocardial infarct using RNA-seq in the Yorkshire pig model [30, 31]. Based on extensive informatics analyses of the transcriptome changes in the DRG and SG tissues followed by validation analyses, we discovered a significant induction of inflammatory signaling and cell death pathway in DRG and SG following acute and chronic myocardial infarction. These findings represent the first unbiased molecular characterization of ANS following cardiac injury, and implicate inflammation and cell death are potential mechanisms involved in ANS remodeling.

## Material and methods

### Animal

Yorkshire pigs (n = 9) were used in this study. All pigs were juvenile males and weighing 48 ± 4 kg at the time of terminal study. Animals undergoing infarction were infarcted at 30 ± 2kg in order to ensure same approximate size as control and acute ischemia animals by the time of tissue harvest. All animal experimental protocols were devised in accordance with guidelines set by the University of California Institutional Animal Care and Use Committee and the National Institutes of Health “Guide for the Care and Use of Laboratory Animals” and approved by the University of California Los Angeles Animal Research Committee (ARC). Animals were housed in the University of California Division of Laboratory Animal Medicine facility. Seven days before the beginning of the experiment, the animals were transported from a farm to the local animal facility where they were individually housed in stalls under conditions of natural light and room temperature. All animals were fed with a commercial grain mixture and tap water ad libitum until 24 hours before surgery. This study followed The ARRIVE Guidelines Checklist for Animal Research: Reporting In Vivo Experiments (S1 File).

### Tissue collection

Animals were randomly assigned to control (n = 3), acute ischemia (n = 3), or chronic infarct with healed left ventricular anteroapical infarct (n = 3). Neural tissue was harvested from the left and right stellate ganglion as well as, the left and right dorsal root ganglion from thoracic (T1-T4) and lumbar spinal regions (L1-L4). For terminal tissue harvest, animals were sedated with intramuscular telazol (4-6mg/kg), intubated and mechanically ventilated. General anesthesia was maintained with inhaled isoflurane (1.5–2.5%) and intravenous boluses of fentanyl (total 10–30μg/kg) during surgical prep. Heart rate and surface 12-lead ECG were monitored throughout using standard limb lead electrocardiogram. The femoral artery was catheterized to monitor the arterial blood pressure and the femoral vein was catheterized for intravenous saline infusion (10 mL/kg). Hourly arterial blood gas was tested and adjustments via ventilation or infusion of sodium bicarbonate were performed as necessary to maintain acid-base homeostasis. In the supine position, animals underwent median sternotomy to expose the heart as well as the left and right stellate ganglion. Animals were then placed prone and a dorsal spinal laminectomy was performed to expose the T1-T4 and L1-L4 dorsal roots bilaterally. Bilateral stellate ganglion, thoracic dorsal root ganglion, and lumbar dorsal root ganglion tissue samples were obtained. Animals were euthanized by terminal cardiac arrhythmia under deep anesthesia. All experiments were performed during the day and tissue collection was obtained between 2 and 3pm.

### Chronic myocardial infarction

Myocardial infarction was created as previously described[32]. Briefly animals were sedated with intramuscular telazol (4-6mg/kg), intubated and mechanically ventilated. General anesthesia was maintained with inhaled isoflurane (1.5–2.5%) and intravenous boluses of fentanyl (total 10–30μg/kg) during surgical prep. A 12-lead electrocardiogram and arterial pressure were monitored. After femoral artery access was obtained, a guidewire (0.035-inch Amplatz Super Stiff Guidewire with J-Tip; Boston Scientific, Marlborough, MA, USA) was placed into the left main coronary artery with fluoroscopy guidance. Over the guidewire, a 3 mm angioplasty balloon catheter (FoxCross PTA Catheter; Abbot Vascular, Temecula, CA, USA) was advanced and inflated at approximately the third diagonal coronary artery arising from the left anterior descending coronary artery. After balloon inflation (30 seconds) a 5 ml suspension of saline containing 1 ml polystyrene microspheres (Poly- bead, 90 μm diameter; Polysciences Inc., Warrington, PA, USA) was injected distally into the artery through the central lumen of the catheter. Occlusion of the artery was visualized by contrast angiography, and acute myocardial infarction (MI) was confirmed by the presence of ST-segment elevations (0.1mv above baseline) in ≥ 5 contiguous limb and precordial leads. Animals recovered post-MI for 6–8 weeks prior to terminal experiments. All animals had epicardial echocardiograms performed prior to terminal experiments which demonstrated left ventricular anteroapical infarcts as defined by regional hypokinesis in the anterior, anteroseptal, and apical left ventricular walls and a reduction in ejection fraction of ≥ 20% from baseline.

### Acute myocardial ischemia

In an open chest, a 4–0 prolene suture was placed around the 2^nd^ diagonal branch of the left anterior descending (LAD) coronary artery. Ischemia was induced by coronary artery ligation for 15 minute followed by reperfusion for 3 hours following coronary artery ligation removal. During coronary artery ligation, ischemia was confirmed by the presence of ST-segment elevations (0.1mv above baseline) in ≥ 5 contiguous limb and precordial leads as well as by direct visualization of the heart in the open chest. During ischemia regional hypokinesis of the left ventricular anteroapical walls distal to the ligation site was observed within 1 minute of coronary artery ligation.

### RNA-Seq

RNA was extracted from T1-T4 dorsal root ganglia and stellate ganglia in different treatment groups of Yorkshire pigs (control n = 3, acute ischemia n = 3, and chronic infarct n = 2) using Trizol reagent (Thermo Fisher Scientific) according to the manufacture’s protocol. RNA sequencing was performed as previously described [33, 34].

### Transcriptome analysis

**Fig 1:**
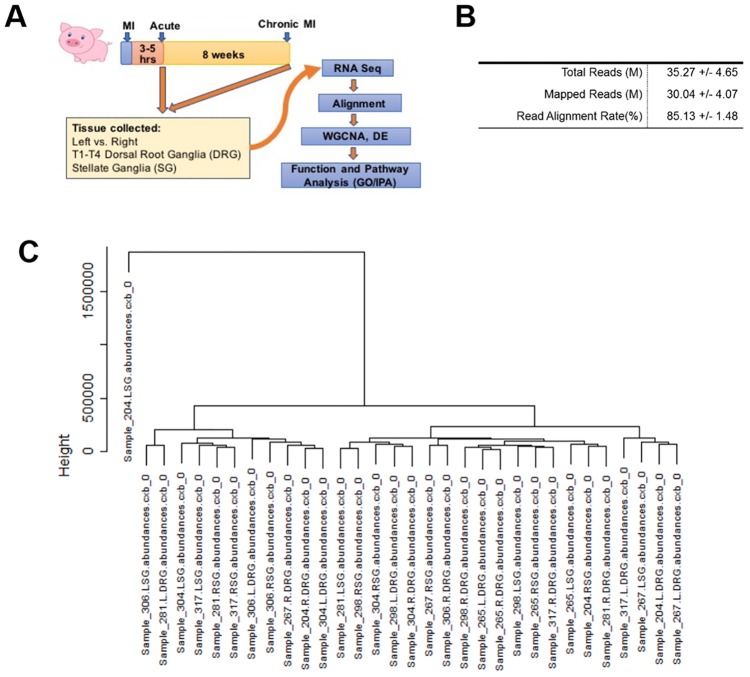
Overview of experimental design and gene expression profiling in stellate ganglia and dorsal root ganglia post myocardial infarct treatment. **A**: Schematic view of experimental design. Yorkshire pigs were treated either with acute MI (3–5 hours) or chronic MI for 8 weeks before tissue collection. (T1-T4) Dorsal root ganglia and stellate ganglia were subjected to RNA-Sequencing. The seq reads were mapped to the pig transcriptome and differentially expressed genes were annotated. **B**. Overview of RNA-seq Alignment: Alignment statistics for the 32 samples used in this study **C**. RNAseq samples were clustered using hclust in R and the 204 LSG Sample was removed as an outlier.

Transcriptomes were analyzed using the tuxedo suite of tools. Briefly, bases with poor quality scores were eliminated from the analysis using the fastq_quality_trimmer tool, and reads with fewer than 50 remaining bases were removed from the analysis. Remaining reads for each sample were mapped to the Sscrofa10.2 genome using Tophat 2.1.1 and counted, annotated and compared using cufflinks 2.2.1. Hierarchical Clustering was used to determine that one sample (204 LSG) was an outlier with no similarity to any other sample (Fig 1C), and it was removed from further analyses. Left and right SG/DRG were treated as individual samples. In all cases an FDR of 5% was used. Cuffdiff was used to calculate statistical differences coming from RNA-seq as previously described[35].

### Weighted gene co-expression network analysis (WGCNA)

WGCNA is a well-documented and commonly used algorithm for determining clusters of co-expressed genes which share functional roles and are linked to phenotypic traits of interest. Transcriptomes were filtered for all genes which were expressed (FPKM >1) and varying (CV >5%) across the 31 high-quality genomes (9589 genes). A correlation matrix was generated comparing each gene to the expression of each other gene, and, per the recommendations of the package, this matrix was transformed into an approximately scale-free (fit = 60%) network by raising the correlation network to a power (7). Topological overlap between each pair of genes is determined by the following equation:

$$TOM_{ij}=\frac{\sum_{u}a_{iu}a_{uj}+a_{ij}}{\text{min}\left(k_{i},k_{j}\right)+1-a_{ij}}$$

where a is the correlation matrix raised to the power of 7, and k is the total number of edges for genes i or j. This matrix is then subjected to hierarchical clustering and a dynamic module detection algorithm creates a first set of possible modules.

The eigengene, the first principle component of each module, is calculated, and modules with high similarity (R>0.75) are merged to identify the final set of modules of interest.

### GO enrichment

Module lists were given to the GeneAnalysis package (ga.genecards.org) which uses a binomial distribution to test the null hypothesis that the genes within the module are not overrepresented in a given category.

### TUNEL staining

TUNEL staining was performed in healthy control, acute myocardial ischemia and chronic myocardial infarct treated stellate ganglia tissue sections using Click-iT Plus TUNEL Assay, Alexa Fluor 488 dye (Thermo Fisher Scientific, C10617) according to the manufacture’s protocol. Nucleus was visualized by Hoechst 33342 (Thermo Fisher Scientific, H3570).

### Immune-staining

Immune-staining was performed in healthy control (n = 3), acute myocardial ischemia (n = 3) and chronic myocardial infarct (n = 3) treated stellate ganglia tissue section. Briefly, the tissue sections was deparaffinized and the antigen was unmasked using citric acid before blocking with blocking buffer (1*PBS/1%BSA/0.3%Triton X-100) at room temperature for one hour followed by incubation with tyrosine hydroxylase antibody (Abcam, ab 112) at 4 degree over night. The tissue sections were then rinsed with PBS for 3 times before incubating with anti-rabbit secondary antibody (ThermoFisher Scientific, R37117) for one hour at room temperature before staining with Hoechst 33342 (Thermo Fisher Scientific, H3570).

### Quantification of mRNA by qRT-PCR

1μg RNA was used for first-strand cDNA synthesis using Random Primer and ProtoScript II Reverse Transcriptase (New England BioLabs, M0368L) according to manufacturer’s instruction. Real-time PCR (Table 1) was performed using IQ SYBR Green Supermix (Bio-Rad) with CFX-96 Real-time PCR Detection System (Bio-Rad). Quantification of mRNA was calculated from on-board program using 18s rRNA as a reference gene for normalization.

**Table 1:** Primers for Real-time PCR.

Primer Name	Sequence
pCXCL10 F	CTGTTCGCTGTACCTGCATC
pCXCL10 R	GGATTCAGACATCTTTTCTCCCC
pNPY F	ACCTGGCCTTCTCTGACTTC
pNPY R	AGATGACCACAATCCCCAGG
pPLAU F	ATCAGAGAAGACCCTGGTGC
pPLAU R	CAATCTTAAAGCGGGGCCTC
pID1 F	CTCCCCTACTCGACGAACAG
pID1 R	CAACTCCAGGTCCCAGATGT
pKLHL18 F	TCCCTGAATGTGGTGGAAGT
pKLHL18 R	GTGTCCGTCTCTGGGTTGTA
pMAPK3 F	TCAACATGAAGGCCCGAAAC
pMAPK3 R	CTTCCTCCACTGTGATCCGT
pMCL1 F	CGCAGTAATCGGACTCAACC
pMCL1 R	CAATCCTGCCCCAGTTTGTT
pBCL2A1 F	ACTGGGGAAGGATTGTGACC
pBCL2A1 R	CAAAGCCATTTTCCCAGCCT
pBIRC2 F	AAAGCTGCCTTGGAAATGGG
pBIRC2 R	TAGGAAGCACGCATGTCAAC
pCASP3 F	TGGAAGCAAATCAGTGGACTC
pCASP3 R	CCCTGAGATTTGCAGCATCC
pPARP 1 F	GCTTTGTTCAGAACCGGGAG
pPARP 1 R	AGGGCCTTTTCGAGCTTACT
pTNFRSF1B F	ACTGATCGTGGGTGTGACAG
pTNFRSF1B R	CAGGACCGGGGACACTTC
pPIK3C2B F	GCTATGAGTTTGGCAGCCTC
pPIK3C2B R	TGGTGGTTCATGTGAGTCCA
pRELA F	TCAAGATCTGCCGGGTGAAT
pRELA R	GAACACGATGGCCACTTGTC
pOAS1 F	GACCTCGTCGTCTTCCTCAC
pOAS1 R	GGCAGGACATCAAACTCCAC

### Statistical analysis

Data are expressed as mean± SD. For comparison between two groups, differences were analyzed by 2-tailed Student’s t test. For comparison of multiple groups, differences were analyzed by 1-way ANOVA. Associations between binary phenotypic variables and eigengenes were calculated using the Wilcoxson Rank Sum test. P values ≤ 0.05 were considered as significant.

## Results

The left and right stellate ganglia (SG) and T1-T4 dorsal root ganglia (DRG) tissues were collected from untreated healthy Control, Acute MI (3–5 hours from MI), Chronic MI (8 weeks after MI) pigs. There were no adverse events or unexpected animal mortality with acute ischemia or chronic infarct protocols. From these tissues, total RNAs were prepared and T1-T4 left vs. right DRG samples were pooled. A total of 8 pigs were analyzed including Control (n = 3), from Acute Ischemia (n = 3), and from chronic MI group (n = 2), respectively (Fig 1A). For each animal, 4 total RNA samples of pooled T1-T4 DRG and SG from left or right were used for RNA-seq. The average number of RNA-seq reads and successful mapping rates from the 32 RNA-seq samples were listed (Fig 1B). Clustering analysis showed one left SG sample from Chronic MI was an obvious outlier, likely due to unknown errors in sequencing procedure, and therefore was excluded from further analysis (Fig 1C).

**Fig 2:**
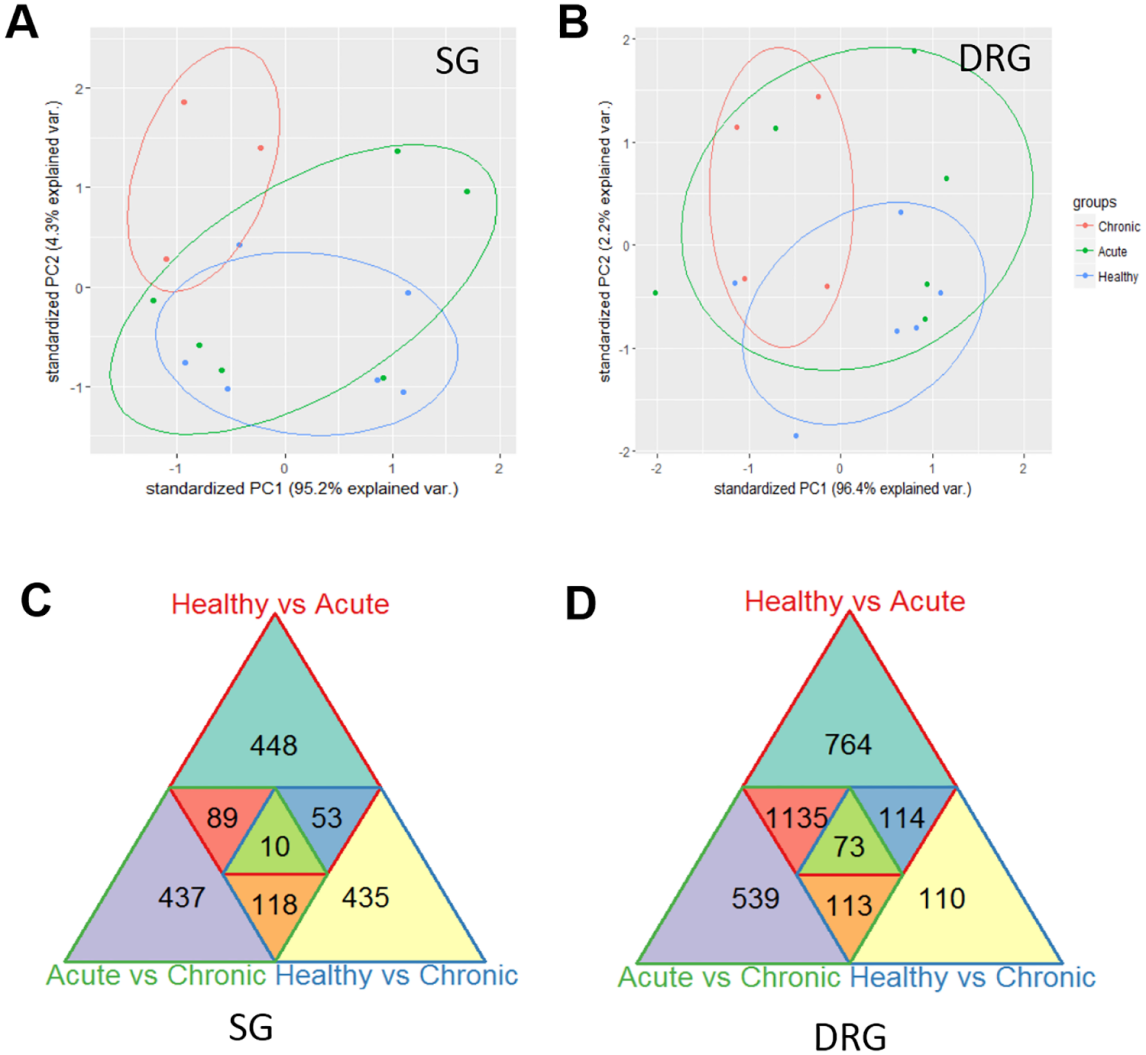
Overview of differential gene expression in stellate ganglia and dorsal root ganglia post myocardial infarct treatment. **A-B**. Principle Component Analysis of SG and DRG Samples. PCA was performed on SG (A) and DRG (B) Samples. In neither case does a single principle component reliably segregate the three treatment groups from one another. **C-D**: Overlap of Differentially Expressed Genes. Differentially expressed genes in either the SG (C) or DRG (D) are displayed, as well as overlaps between multiple categories.

In order to demonstrate the extent of transcriptome remodeling in SG and DRG following acute ischemia or chronic MI, we performed principal component analysis among the SG and DRG samples (Fig 2A and 2B). While marked separation of transcriptome patterns was observed between chronic MI and healthy controls, the acute ischemia transcriptomes showed marked overlap with both chronic MI and healthy controls, implicating more heterogeneous gene expression profile during acute response to ischemia. We also performed pair-wise differential gene expression analysis within each tissue type based on their treatment conditions. As shown in Fig 2C and 2D, we identified large numbers (from 110 to 764) of genes with their expression levels significantly changed in SG or DRG following either acute ischemia or chronic MI. In addition, there are 437 and 539 genes identified to be differentially expressed between acute ischemia and chronic MI samples in SG and DRG, respectively. These data suggest a rapid and heterogeneous global transcriptome remodeling in both SG and DRG in response to cardiac ischemic injury, and this reprogramming continues to progress in chronic phase.

### Inflammation and cell death genes are enriched in transcriptome reprogramming associated with myocardial infarction

**Fig 3:**
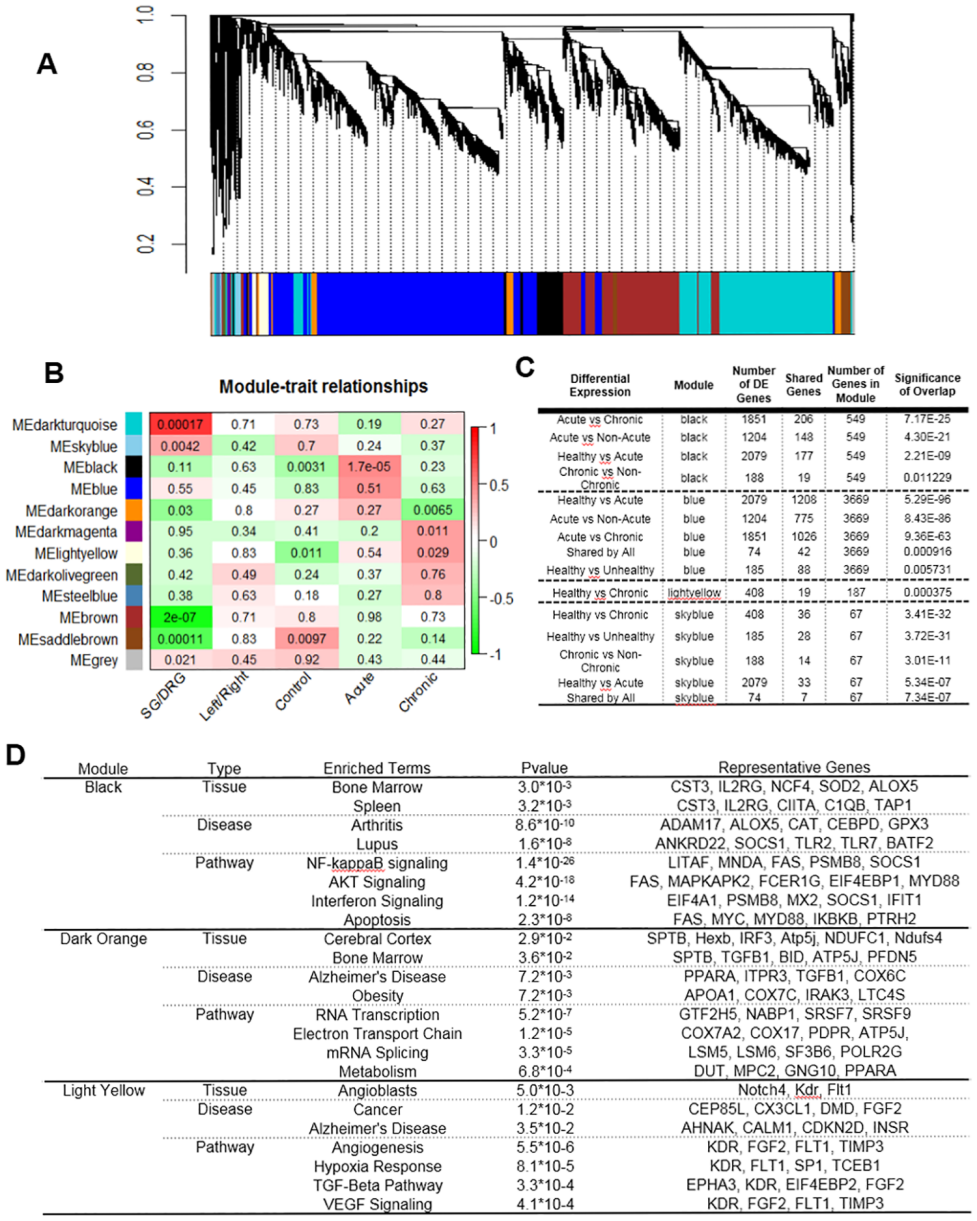
WGCNA revealed differential gene expression enrichment in SG and DRG sample post cardiac stress. **A**: WGCNA performed on expressed and varying genes in the RNA-seq samples resulted in 12 modules of highly correlated genes. **B**: Association of module eigengenes to phenotypic categories reveals significant associations between specific modules and phenotypes. Color indicates direction of association (red positive, green negative), while then number in the box is the Wilcoxson Rank Sum P value. **C**: Significant Overlaps Between DE Genes and Modules. Four modules (of 12) had significant overlaps with differentially expressed genes. **D**: Gene Ontology Enrichment Analysis for different module.

In order to uncover biological relevance of the transcriptome reprogramming observed in the ganglia samples, we performed unsupervised Weighted Gene Co-expression Network Analysis (WGCNA) from differentially expressed genes in order to identify gene modules based on shared expression profile among the 31 RNA-seq samples. As shown in Fig 3A, 12 modules of highly correlated genes were identified. Analysis of module eigengenes for correlation with phenotypic categories revealed that two gene modules were significantly associated with different tissue types (the Dark Turquoise and the Brown module for SG vs. DRG) (Fig 3B), while no gene modules were identified to distinguish left vs. right tissues. Importantly, the Black module was significantly associated with acute ischemia while the Dark Orange and two other modules were significantly associated with Chronic MI (Fig 3B) The Black modules and the light Yellow module member genes were significantly represented by differentially expressed genes identified in acute ischemia or chronic post-MI tissues. These results suggest that the Dark Turquoise and the Brown modules contribute to the tissue identity between SG vs. DRG while the Black module best represents the acute transcriptome changes triggered by myocardial ischemia, and the Dark Orange, Dark Magenta and Light Yellow modules each contributes significantly to the transcriptome signature in chronic MI tissues (Fig 3C). From Gene-Ontology Enrichment analysis, both Dark Turquoise and Brown modules are significantly enriched with genes involved in neural function, consistent with distinct neural function of SG and DRG. In contrast, the Black module contain a large number of genes that are overwhelmingly dominated by genes associated with inflammation and apoptotic cell death (Fig 3D). In addition, the Dark Orange, the Dark Magenta and the Light Yellow modules are enriched by metabolism, cell death and neural function genes. These data implicate distinct molecular signatures associated with acute and chronic reprogramming in SG or DRG following MI (Fig 3D).

### Validation of cellular and molecular changes in autonomic nervous tissue after myocardial infarction

**Fig 4:**
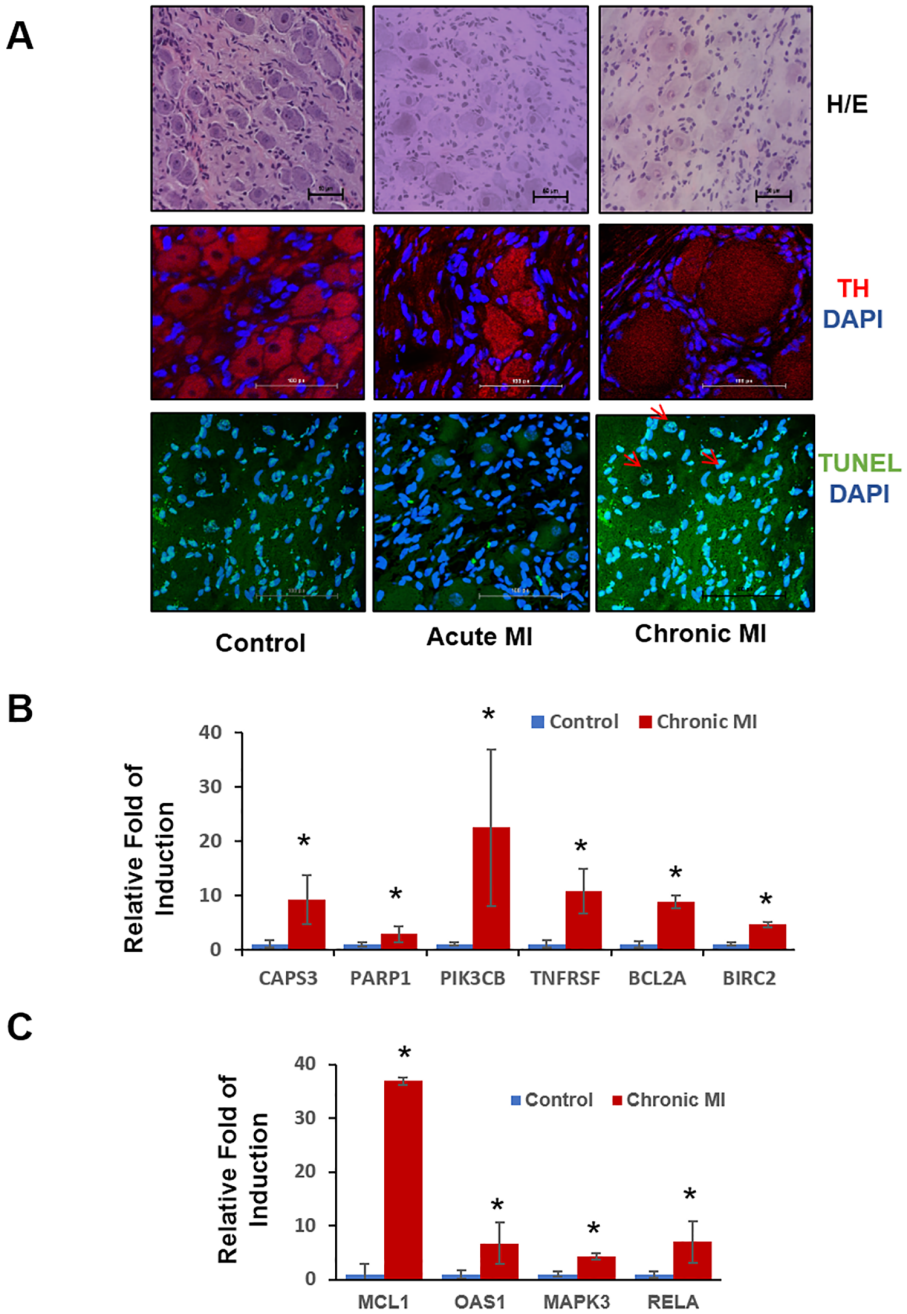
Cell death and apoptosis pathway is significantly activated in chronic MI treated stellate ganglia. **A**: Histological analysis of different groups of stellate ganglia. Top: H&E staining of healthy control; acute ischemia and chronic MI treated stellate ganglia. Magnification 20*. Middle: Neuron body TH expression level detected by immune-staining. Red: TH; Blue DAPI Magnification 40* Bottom: Cell apoptosis detected by TUNEL staining in control, Acute MI or chronic MI treated stellate ganglia. Green, TUNEL; Blue: DAPI. Magnification 40*. **B**: Real-time PCR analysis of cell death pathway gene expression profile in control and chronic MI treated stellate ganglia. n = 3 each sample, *, *p*<0.05 **C**: Real-time PCR analysis of inflammatory pathway gene expression in control and chronic MI treated stellate ganglia. n = 3 each sample, *, *p*<0.05.

To further validate the involvement of apoptotic cell death in the autonomic nervous circuit following MI, we performed histological analysis in the stellate ganglia tissue obtained from Control, acute ischemia and chronic MI groups. Fig 4A showed that more TUNEL positive cells were observed in stellate ganglia from acute ischemia pigs, and an even more dramatic induction of TUNEL positive cells were detected from chronic MI pigs (S1 Fig). In addition, tyrosine hydroxylase (TH) positive neurons were enlarged in sizes following ischemia based on immunostaining signal, particularly in chronic post-MI samples. Furthermore, we directly examined the expression of selected genes in SG and DRG using quantitative RT-PCR.

**Fig 5:**
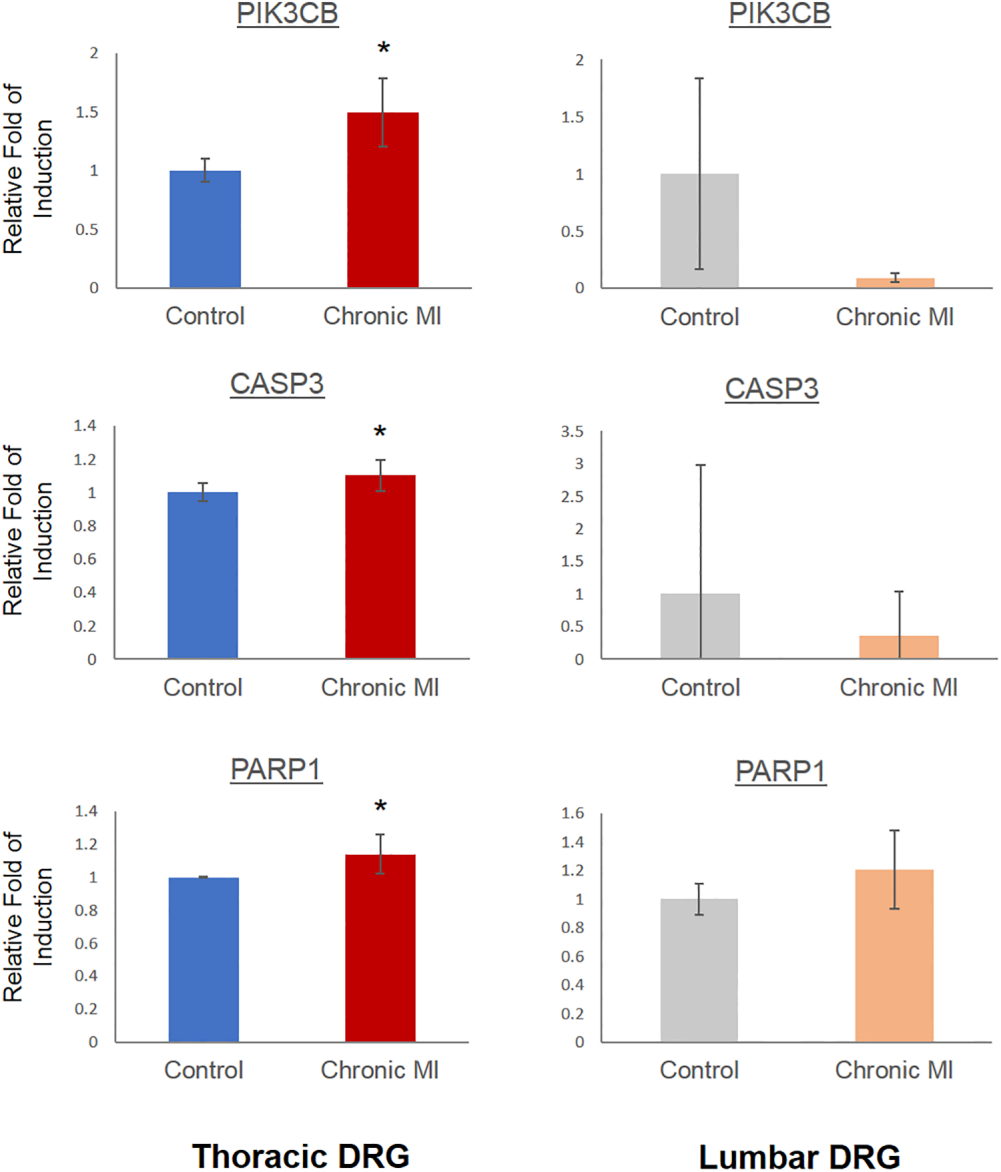
Cell death pathway is activated in thoracic section of chronic MI treated dorsal root ganglia. Real-time PCR analysis of gene expression in thoracic DRG and Lumbar DRG. n = 3 each group, *, *p*<0.05.

**Fig 6:**
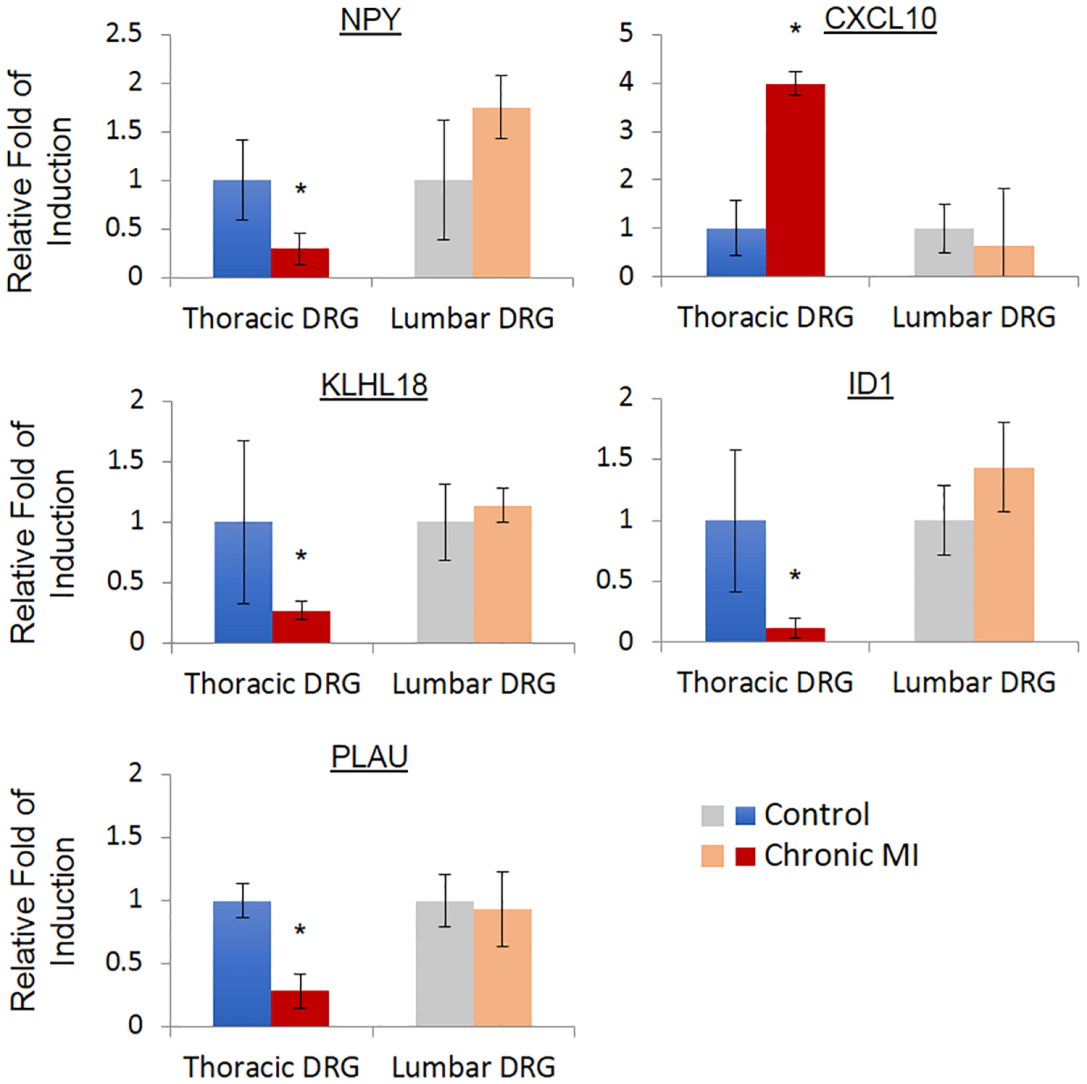
Immune and neuron modulators are changed in chronic MI treated dorsal root ganglia. Real-time PCR analysis of immune and neuron modulators expression in control and chronic MI treated DRG. n = 3 each group, *, *p*<0.05.

As shown in Fig 4B and 4C, the induction of cell death related genes, including Caspase 3 (CASP3), poly (ADP-ribose) polymerase 1 (PARP1), B cell leukemia/lymphoma 2 related protein A1a (BCL2A), Baculoviral IAP repeat containing 2 (BIRC2), Phosphatidylinositol-4,5-bisphosphate 3-kinase catalytic subunit beta (PIK3CB) and TNF receptor superfamily member 1B (TNFRSF) were observed in the SG samples obtained from chronic infarct pigs compared to the SG from untreated Controls. Similar to the cell death regulators, inflammation related genes including Mitogen-activated protein kinase 3 (MAPK3), BCL1 family apoptosis regulator (MCL1), 2,-5-oligoadenylate synthetase 1 (OAS1) and RELA proto-oncogene (RELA) were markedly induced. In order to investigate if the observed induction of inflammatory and pro-death gene expression was a result of systemic inflammatory response to MI injury in heart, we also compared gene expression in thoracic DRG vs. lumber DRG in the chronic MI animals. We observed significant induction of apoptotic genes including PARP1, CASP3 and PIK3CB in the thoracic DRG following MI, but not in the lumbar DRG from the same post-MI animals (Fig 5). Similarly, we observed significant changes in the expression of several neural and immune modulators, including Neuropeptide Y (NPY), C-X-C motif chemokine ligand 10 (CXCL10), Kelch like family member 18 (KLHL18), Inhibitor of DNA binding 1 (ID1) and Plasminogen activator (PLAU) in the thoracic DRG from infarcted pigs relative to the Controls. However, these genes were not altered in the corresponding neurons from lumbar DRGs of the same animals (Fig 6).

## Discussion

An essential element of improving our understanding of neural triggers for cardiac arrhythmias requires identifying the molecular changes in the ANS structural components in response to pathological stress in heart. In this study we employed RNA sequencing technique in a porcine MI model to establish the gene expression changes in stellate ganglia (SG) and dorsal root ganglia (DRG). Our major findings include; 1) global change in gene expression in stellate ganglia and dorsal root ganglia is seen in both acute myocardial ischemia and following chronic myocardial infarction, 2) using weighted gene co-expression network analysis (WGCNA) tool, we also identified specific gene modules expressed in the ganglia that are significantly associated with MI injury in heart, 3) both gene modules and differentially expressed genes are significantly enriched with inflammation and cell death regulators. To our knowledge, this is the first unbiased approach to profile and characterize global molecular changes in ANS following cardiac injury. In addition to the previously known changes in individual genes, such as NPY (Fig 6), our results show a rapid onset of global gene expressions change involving inflammatory and cell death signal activation.

In this study, we observed strong induction of inflammatory genes after acute ischemia and highly elevated apoptotic activity in the extrathoracic SG after chronic myocardial infarction. These data suggest a potential pathogenic process where cardiac injury induces early onset of inflammatory gene expression in ANS neurons, which is followed by apoptotic cell death and abnormal activities. Our gene expression findings of neural remodeling in the SG following myocardial injury are supported by previous studies in both animal and clinical models.[29, 36–38] Han et al demonstrated increased SG nerve densities and neural activity after only 1-hr of ischemia in a canine model.[29] While in humans, transdifferentiation between cholinergic and adrenergic neurons and changes in expression of certain factors including neuronal nitric oxide synthase have been demonstrated in SG after myocardial infarction.[15, 32]

Our study expands on the results from previous investigations that have demonstrated neuronal cellular changes after ischemia. In this study, we describe the global genetic changes which are thought to be underlying the observed neural remodeling with inflammatory processes being activated in early ischemia followed by apoptosis and abnormal cell function after chronic infarction. Inflammation has recently been implicated in the pathogenesis of heart failure and as a target for developing potential therapies for acute or chronic heart diseases. The sympathetic and parasympathetic nervous system have been reported as targets for treating inflammatory states like myocarditis[39]. Although much of the inflammation induction in heart failure has been investigated in post-stress myocardium, a direct role of inflammation in ANS remodeling has not been fully demonstrated. One of the major consequences of inflammatory induction in neurons is over-sensitization and over-activity, leading to cell death. Although our current study did not directly modulate the inflammatory process, histological observation in chronic post-MI animals revealed profound neural hypertrophy associated with elevated apoptosis supporting the mechanistic link between cardiac injury and ectopic inflammation (Fig 4A).

While neural remodeling in the intrathoracic sympathetic ganglia (SG) following myocardial ischemia has been hypothesized to be caused by local mechanisms such as retrograde axonal transport of proteins from infarcted myocardium [37], mechanisms of molecular changes in the central neuraxial components of the afferent limb, relay and processing centers in the extra-cardiac central sympathetic nervous system such as, the dorsal root ganglion and spinal cord, are unknown. Therefore, in this study we sought to also investigate the genetic changes in the afferent limb of neural signaling from ischemic myocardium to thoracic DRG.

We found changes in gene expression localized to thoracic spinal cord DRG only, with no change in gene expression in lumbar DRG, after myocardial ischemia or infarction. Significant changes in the expression of several neural and immune modulators (NPY, CXCL10, KLHL18, ID1 and PLAU) and apoptotic genes (PARP1, CAPS3 and PIK3CB) were observed in the thoracic DRG following MI, but not in the lumbar DRG from the same post-MI animals. The fact that both inflammation and cell death signals were only observed in the ganglia tissues from thoracic DRG but not from the lumbar DRG suggests that these changes are specifically related to sympathetic ANS circuit innervating heart rather than systemic effects associated with MI. These finding suggest that cardiac arrhythmias following myocardial injury may in part be due to neural remodeling occurring throughout neural circuits in the intrinsic and extrinsic cardiac nervous system, thus leading to ANS imbalance and increased sympatho-excitation.

## Limitations

Our current transcriptome profiling was limited by the tissue heterogeneity and the cell-autonomous mechanism associated with the observed induction in inflammation and cell death. Due to the small sizes, the collected tissues may have been contaminated by surrounding muscle or fat tissues which may have contributed to heterogeneity observed in the gene expression profile. In addition, the histological analysis did not include sufficient information to determine the cellular type responsible for inflammatory gene expression or apoptotic signal. Further studies with more resolution and cellular identification information will be needed to fully illustrate the mechanistic contribution and therapeutic potential for our findings.

## Conclusions

Myocardial injury leads to time-dependent global changes in gene expression in the innervating ANS. Induction of inflammatory gene expression and loss of neuron cell viability in SG and DRG are potential novel mechanisms contributing to abnormal ANS function that can promote cardiac arrhythmias and pathological remodeling in myocardium. These findings lead to obvious questions for future investigations. Are inflammation and neural cell death associated with sympathetic overdrive? Do neuromodulation therapies ameliorate ANS remodeling and attenuate cardiac arrhythmias or remodeling following injury? Greater understanding of the molecular changes in the ANS following cardiac injury will allow deeper insights into the mechanisms of cardiac arrhythmias and help in the optimization of neuraxial therapies.

## Supporting information

S1 File The ARRIVE Guidelines Checklist. (PDF)

S1 Fig Quantification of apoptotic cells in Fig 4A. (TIF)
