# Supplementary figures and images for: Inflammatory and apoptotic remodeling in autonomic nervous system following myocardial infarction
Source: PLoS One. 2017 May 18;12(5):e0177750. doi: 10.1371/journal.pone.0177750 (PMC5436752; doi:10.1371/journal.pone.0177750)

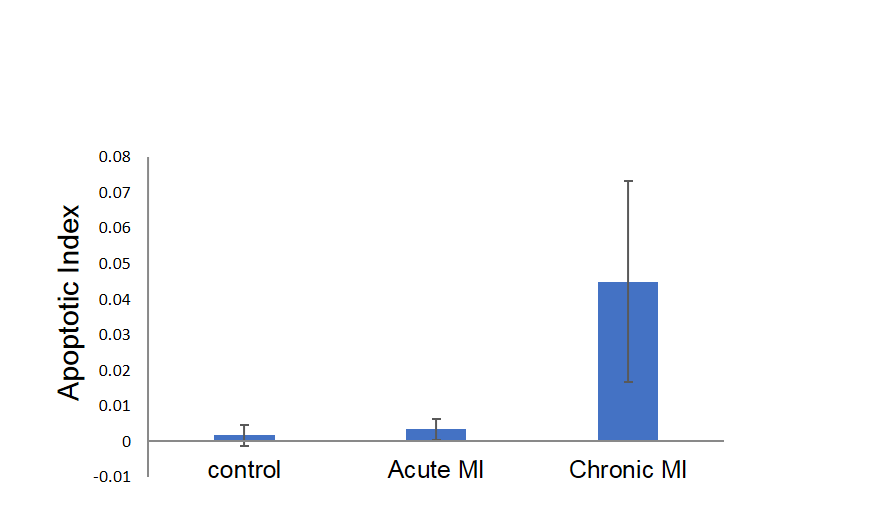

Supplement: S1 Fig — (TIF) [file pone.0177750.s002.tif]
